# Hybrid Silica Xerogel and Titania/Silica Xerogel Dispersions Reinforcing Hydrophilicity and Antimicrobial Resistance of Leathers

**DOI:** 10.3390/gels9090685

**Published:** 2023-08-25

**Authors:** Michael Arkas, Theofanis Bompotis, Konstantinos Giannakopoulos, Evangelos P. Favvas, Marina Arvanitopoulou, Konstantinos Arvanitopoulos, Labros Arvanitopoulos, Georgia Kythreoti, Michail Vardavoulias, Dimitrios A. Giannakoudakis, Laura Castellsagués, Sara Maria Soto González

**Affiliations:** 1 Institute of Nanoscience Nanotechnology, NCSR “Demokritos”, Patriarchou Gregoriou Street, 15310 Athens, Greece; fanisbobotis@gmail.com (T.B.); k.giannakopoulos@inn.demokritos.gr (K.G.); e.favvas@inn.demokritos.gr (E.P.F.); marinaarva3@gmail.com (M.A.); 2 DARVICHEM Alexandrou, Papagou 5, 18233 Agios Ioannis Rentis, Greece; darvichem@hol.gr (K.A.); info@darvichem.gr (L.A.); 3 Institute of Bioscience and Applications, NCSR “Demokritos”, Patriarchou Gregoriou Street, 15310 Athens, Greece; gkythreoti@acg.edu; 4 Department of Science and Mathematics, School of Liberal Arts and Sciences, The American College of Greece, Deree, Gravias 6, 15342 Athens, Greece; 5 PYROGENESIS S.A., Technological Park 1, Athinon Avenue, 19500 Attica, Greece; mvardavoulias@pyrogenesis-sa.gr; 6 Department Chemistry, Aristotle University of Thessaloniki, 54124 Thessaloniki, Greece; dagchem@gmail.com; 7 Barcelona Institute for Global Health (ISGlobal), Universitat de Barcelona, 08036 Barcelona, Spain; laura.castellsagues@isglobal.org (L.C.); sara.soto@isglobal.org (S.M.S.G.); 8 CIBER Enfermedades Infecciosas (CIBERINFEC), Instituto de Salud Carlos III, 28029 Madrid, Spain

**Keywords:** titanium oxide, antibacterial, antifungal, antibiofilm, hyperbranched, dendrimers, polyethylene imine, IR spectroscopy, contact angle, electron dispersion spectroscopy

## Abstract

Four leather substrates from different animals were treated by dispersions containing hydrophilic composite silica-hyperbranched poly(ethylene imine) xerogels. Antimicrobial activity was introduced by incorporating silver nanoparticles and/or benzalkonium chloride. The gel precursor solutions were also infused before gelation to titanium oxide powders typically employed for induction of self-cleaning properties. The dispersions from these biomimetically premade xerogels integrate environmentally friendly materials with short coating times. Scanning electron microscopy (SEM) provided information on the powder distribution onto the leathers. Substrate and coating composition were estimated by infrared spectroscopy (IR) and energy-dispersive X-ray spectroscopy (EDS). Surface hydrophilicity and water permeability were assessed by water-contact angle experiments. The diffusion of the leather’s initial components and xerogel additives into the water were measured by Ultraviolet-Visible (UV-Vis) spectroscopy. Protection against GRAM- bacteria was tested for *Escherichia coli*, *Pseudomonas aeruginosa*, and *Klebsiella pneumoniae* against GRAM+ bacteria for *Staphylococcus aureus* and *Enterococcus faecalis* and against fungi for *Candida albicans*. Antibiofilm capacity experiments were performed against *Staphylococcus aureus*, *Klebsiella pneumoniae*, *Enterococcus faecalis*, and *Candida albicans*. The application of xerogel dispersions proved an adequate and economically feasible alternative to the direct gel formation into the substrate’s pores for the preparation of leathers intended for medical uses.

## 1. Introduction

The hides of killed animals were the obvious and abundant raw material used by humanoids for protection against cold and other adverse environmental challenges. As a consequence, the development of their treatment dates to prehistory. Nowadays applications do not restrict to clothing and relevant accessories but also extend to fields such as bookbinding, car and furniture upholstery, and most importantly medicine. Implementations in the latter area include wheelchair lining, surgical ICU and examination beds, medical shoes, leather anti-decubitus pillows, and hospital chairs. Advanced functionalities and properties are needed for the fulfilment of these roles including resistance to microbe contamination and biofilm formation combined with elevated water permeability. For this reason, a complex chemical treatment is required for the transformation of raw hides into appropriate medicinal leathers. This comprises liming, deliming, bating, and multi-stage tanning procedures for antisepsis that secure antibacterial and antifungal protection and dying. During the finishing process, various additional special coatings are applied to provide characteristic odor, coloring, abrasion resistance, and antiallergic properties [1]. Toxic solvents and substances, for instance, hexavalent chromium, formaldehyde, azo-dyes sodium sulfite, chlorinated paraffin, heavy metals, and perfluorinated compounds are involved. All these chemicals cause severe environmental impacts [2,3] and the need therefore for more environmentally friendly treatments is imminent.

It is well known that silica gels are widely applied in the fields of chemical compound separation and purification by chromatography and in the pharmaceutical industry as fillers or protracted release drug carriers [4,5]. They are perhaps the most common desiccants or moisture indicators and may also serve as additives for foods and dyes. They are typically produced from the polymerization of silicates or orthosilicic acid which initially affords hydrogels. Slow water evaporation transforms unreacted silanol groups into siloxanes and grants xerogels with large surface area, high porosity, and small pore sizes. The latter have been proven biocompatible, versatile, and more effective in hosting substances sensitive to humidity or oxidation [6,7].

The use of silver nanoparticles (AgNPs) as an effective microbicide alternative to chromium is extensive due to their broad spectrum [8,9,10]. They are highly reactive due to their large surface-to-volume ratio and play a key role in inhibiting bacterial growth in aqueous and solid media. Therapeutic applications include catheters [11] and wound dressings [12]. Several studies have reported the application of AgNPs to leathers as colloidal solutions and emulsions [13,14] and through microencapsulation [15].

Unlike textiles, leather cannot be washed conventionally in washing machines. To address this problem, modern finishing techniques contemplate self-cleaning surface coatings. In this field, the most commonly used method is super hydrophilic layers that prevent the deposition of stains. Titanium dioxide TiO_2_ is known for its inherent hydrophilicity and distinctive photocatalytic capacity and is perfectly suitable for this purpose. In this context, TiO_2_ nanoparticles were prepared, incorporated into acrylic binding plasters, and applied to the surface of skins through a finishing process [16]. The increase in the leather surface’s hydrophilicity enhances the photocatalytic properties and the decomposition of organic residues and stains. In another implementation, casein, polyacrylates, and commercially available TiO_2_ NPs were incorporated into a composite membrane that presented effective self-cleaning ability on stains such as coffee, red wine, paint, and oil [17]. Stain decomposition is attributed to induced oxidative degradation from the generated active radicals.

Titanium dioxide is used additionally in various antimicrobial practical applications, such as water and air purification and self-cleaning and self-sterilizing surfaces [18,19,20,21]. It has been reported that silver-doped TiO_2_ inhibits the growth and proliferation of microorganisms at very low concentrations [22]. It constitutes a suitable matrix for a TiO_2_-Ag composite antibacterial agent because AgNPs tend to present a good distribution inside titanium dioxide [23]. Furthermore, silver improves the bioactivity of titanium dioxide TiO_2_ [24]. Therefore, the combination of AgNPs and TiO_2_ could lead to improved properties.

The thriving class of dendritic macromolecules is the outcome of radical polymerization [25]. Due to this ever-increasing scientific interest and completely different chemical behavior in comparison to the other conventional macromolecules, they are separated from their linear, crosslinked, and branched counterparts and categorized as the fourth major class of polymers [26,27,28,29,30,31]. The tree-reminiscent structure characterized by the repetitive branched motif induces a collection of useful properties. Perhaps the most important is extreme versatility as the three major architectural parts consisting of internal cores, branching points, and external functional groups are readily convertible via conventional synthetic paths to adapt to the desired scope [32,33]. Inner branches particularly form cavities that may host active ingredients. Quaternary ammonium salts characterized by their organization that leads to high local concentrations of cations present antimicrobial properties [34,35]. Amongst them, a mixture of C8 to C18 alkyl benzyl dimethyl ammonium chlorides produce benzalkonium chloride (BAC) which since 1935 [36] has been a common commercial surfactant biocide that is applied in textiles [37,38] and presents an attractive option as a guest. The internal pockets of hyperbranched poly(ethyleneimine) (PEI) are characterized by high hydrophilicity and density of lone pairs rendering this dendritic macromolecule a suitable carrier. Furthermore, PEI presents an endogenous bactericide activity that in synergy with the active ingredient produces an additive effect [39].

Another noteworthy element is the ability of dendritic polymers to act as microreactors. Reactions performed into the cavities formed by the inner branches or the periphery are not limited by the standard rules of solution chemistry. Restricted medium and polyvalency effects promote procedures inspired by biological paths. In this context, the incorporation of metal ions into the dendritic pockets yields metal nanoparticles by a process similar to biomineralization [40]. External functional groups such as amines may mimic the effect of proteins like silaffins and form ceramic shells [41,42,43,44,45]. When these two methods are merged, the outcome is composite inorganic (ceramic)-organic (polymer matrix)-inorganic (metal) nanoparticles and gels with distinguishing physicochemical properties. The derived hybrids are exemplary tools for a multitude of applications, for example in catalysis [46,47], water purification [48,49], textiles [50], and medicine [51,52,53]. Furthermore, only ambient temperatures and aqueous solutions are involved so these materials can be considered eco-friendly.

In a recent implementation of the above principles, our group produced Ag Nps by biomimetic mineralization via the mediation of a variety of hyperbranched PEIs. By the addition of orthosilicic acid, silica gel forming reactions were then performed into the pores of leather substrates. This coating formulation unexpectedly dramatically increased hydrophilicity. The resulting substrates could easily adsorb additional active ingredients, i.e., BAC. Furthermore, it prevented the diffusion of valuable leather components into water. The final products demonstrated excellent antibacterial and antiviral properties. Additionally, the interaction of the negatively charged silanol groups of the orthosilicic acid eliminated the toxicity due to local aggregations of positively charged terminal PEI ammonium groups [54]. The drawback of the preparation is that those ecological biomimetic procedures take a lot of time and xerogel precursor solutions must be prepared in situ (Ag Nps aggregate and precipitate if left in solution and gelation usually takes a few hours). This means that industrial upscaling would require large leather treatment reactors to operate for days and thus render the method financially unprofitable. It is the scope of the current work to develop an alternative method and produce simple xerogel powders that comply with the typical leather processing requirements. These will be prepared by chemical companies that produce leather treatment additives. They will then be used to form dispersions. The latter will be directly applied to medical leathers in the reactors during short time intervals. Moreover, in a further adaptation to the industrial standards, combinations of the xerogel precursor solutions with TiO_2_ powders will also be tested to produce coatings with additional advantageous properties.

## 2. Results and Discussion

### 2.1. Sample Preparation

A detailed characterization of simple hyperbranched PEI xerogels with orthosilicic acid as well as of their analogs containing silver nanoparticles is presented in our preceding works [52,54]. As mentioned previously in the introduction, the scope of the current study is to conclude if the application of hydrophilic antimicrobial silica xerogel dispersions by spraying can replace their direct formation into leather pores. Furthermore, xerogel formation in combination with TiO_2_ powder is also investigated. Three titania dispersions comprising silica xerogels of different compositions are characterized and tested. All dispersions were applied on four different substrates. Leathers from three different animals (Buffalo, Cow, Sheep) with different colors and finishing recipes were employed. All samples are summarized in Table 1. Detailed information on the synthesis of the silica xerogels containing silver nanoparticles and the silica xerogel and silica xerogel/titania dispersions is included in the experimental section.

### 2.2. Scanning Electron Microscopy

**Figure 1:**
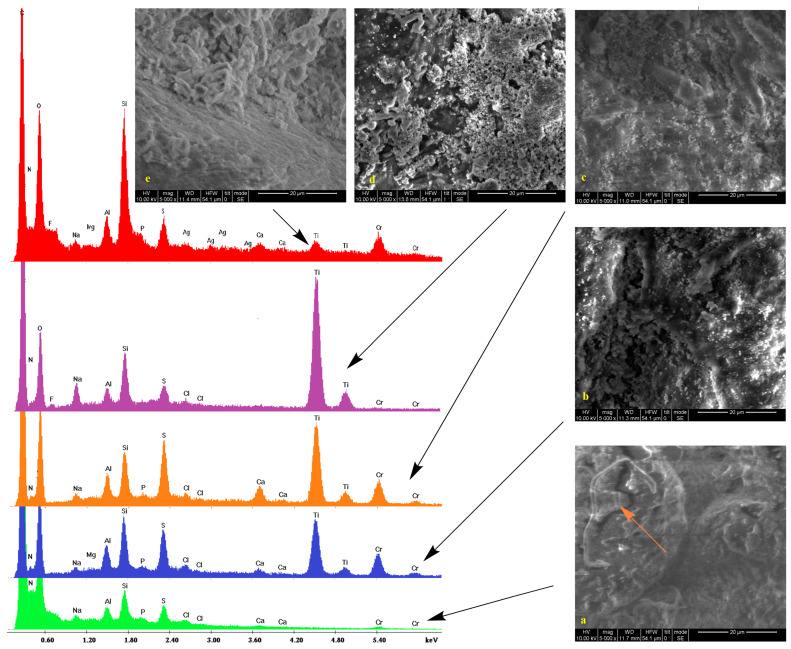
SEM micrographs and EDS spectra of Crust Havane Buffalo leather (**a**, green) treated with Ti-Si-PEI 25,000-BAC (**b**, blue), Ti-Si-PEI 25,000-Ag (**c**, orange), Ti-Si-PEI 25,000-Ag-BAC (**d**, violet), and Si-PEI 25,000-Ag (**e**, red). Orange arrow indicates anomalies (see text).

**Figure 2:**
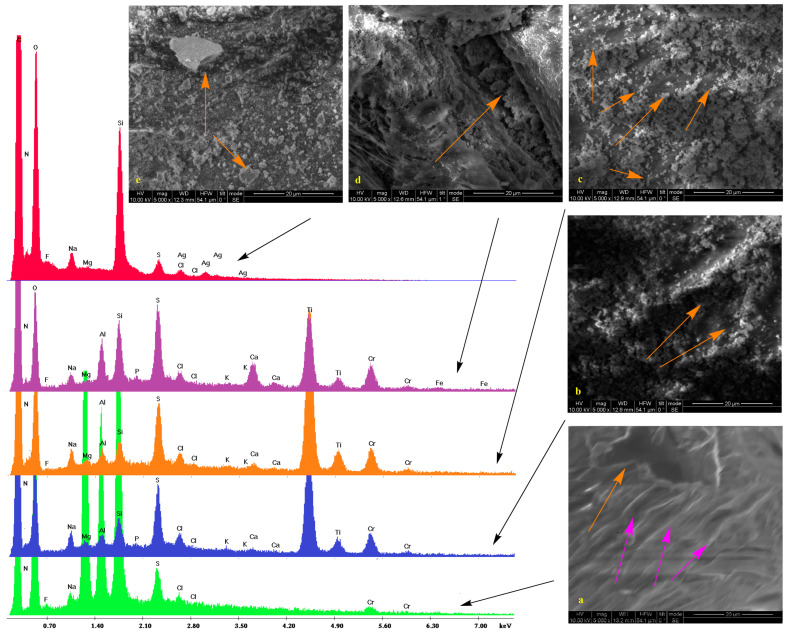
SEM micrographs and EDS spectra of Black Crust Buffalo (**a**, green) leather treated with Ti-Si-PEI 25,000-BAC (**b**, blue), Ti-Si-PEI 25,000-Ag (**c**, orange), Ti-Si-PEI 25,000-Ag-BAC (**d**, violet), and Si-PEI 25,000-Ag (**e**, red). Figure 2a orange arrow indicates a pore; pink arrows show folds. Figure 2b orange arrows indicate coated smaller elevations. Figure 2c orange arrows indicate uncoated flat regions. Figure 2d orange arrow indicates coating material into a pore. Figure 2e orange arrows indicate silica aggregates.

**Figure 3:**
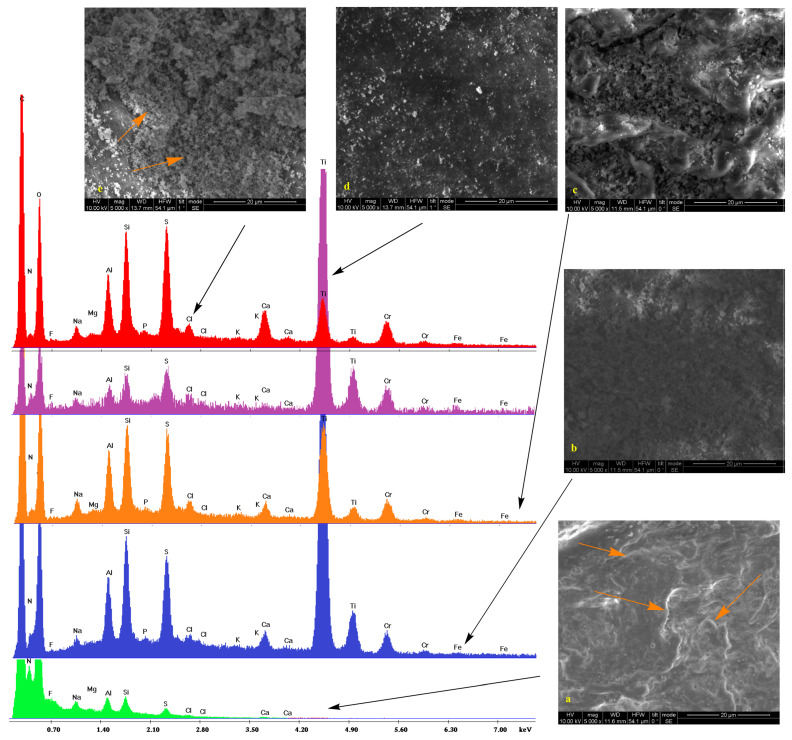
SEM micrographs and EDS spectra of White Crust Cow leather (**a**, green) treated with Ti-Si-PEI 25,000-BAC (**b**, blue), Ti-Si-PEI 25,000-Ag (**c**, orange), Ti-Si-PEI 25,000-Ag-BAC (**d**, violet), and Si-PEI 25,000-Ag (**e**, red) Figure 3a orange arrows indicate the roughness of the raw leathers. Figure 3e orange arrows indicate spherical particles reminiscent of biomimetic silica nanosphere formation.

**Figure 4:**
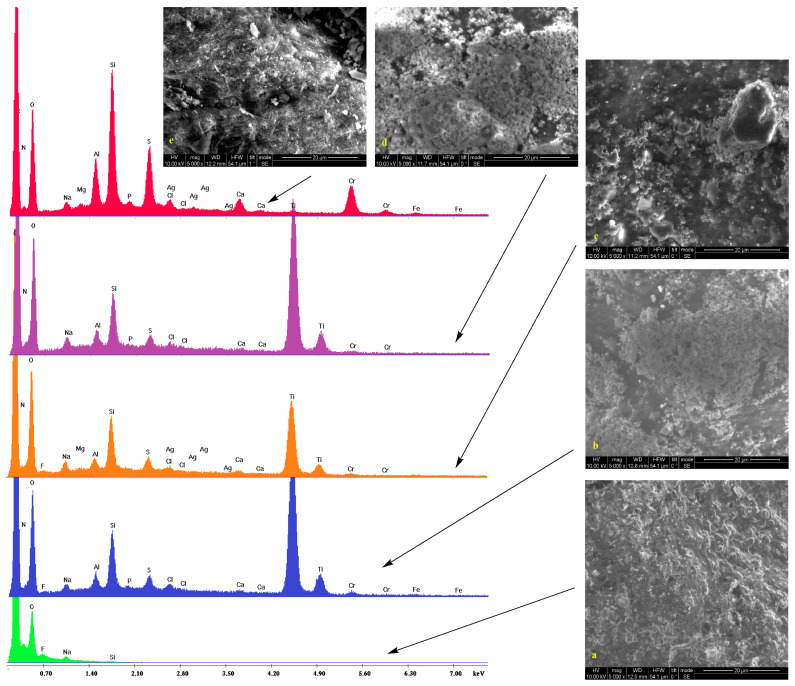
SEM micrographs and EDS spectra of Black Finished Sheep leather (**a**, green) treated with Ti-Si-PEI 25,000-BAC (**b**, blue), Ti-Si-PEI 25,000-Ag (**c**, orange), Ti-Si-PEI 25,000-Ag-BAC (**d**, violet), and Si-PEI 25,000-Ag (**e**, red) dispersions.

Low vacuum SEM micrographs along with the EDS spectra of untreated samples and those treated with silica/titania and silica dispersions (see experimental section) are shown in Figure 1, Figure 2, Figure 3 and Figure 4. In some cases, pores are visible. On the surface of bare substrates, they are distinguishable as darker irregular elliptic formations (orange arrow, Figure 2a). Folds (pink arrows, Figure 2a), other anomalies (orange arrow, Figure 1a), and a general non-uniformity and roughness of the raw leathers (Figure 3a) are also apparent. Treatment with silica/titania dispersions causes the appearance of particle aggregates of quasi-spherical structures that partially cover the leather layer. Empty spaces come into sight in flat regions (orange arrows, Figure 2c), whereas the coating material infiltrates some of the pores (orange arrow, Figure 2d) and also covers the smaller elevations (orange arrow, Figure 2b). More or less the same remarks apply to the silica coatings. The silica aggregates are generally bigger nonetheless (orange arrows, Figure 2e) and spherical particles reminiscent of biomimetic silica nanosphere formation [47,55] are rarer (orange arrow, Figure 3e) since their typical formation by precipitation is inhibited by the gelation procedure.

The pretreatment stages of raw bovine, buffalo, and sheep hides are reflected in the peaks and the corresponding elements of energy-dispersive X-ray spectra. Collagen and the other organic compounds of the untreated leathers are represented by the carbon, nitrogen, and oxygen peaks. Chlorine presence is justified by the NaCl used for the preservation of the initial hides while the traces of fluorine indicate the employment of perfluorinated compounds for induction of water resistance. Magnesium owes its presence to MgO added during the re-tanning. Aluminum and silicon peaks are present due to an alternative reagent, sodium aluminosilicate (AlNa_12_SiO_5_), applied for the same process and kaolinite (Al_2_O_3_ 2SiO_2_·2H_2_O) used during bating. Apart from NaCl and AlNa_12_SiO_5_, sodium is found in Na_2_S that denatures the interfibrillar proteins. Calcium derives from the Ca(OH)_2_ of the liming process with the skin swelling under alkaline conditions for stabilization of the collagen fibers. Sulfur (in addition to Na_2_S) and chromium compose the tanning agent ([Cr(H2O)_6_]_2_(SO_4_)_3_), while potassium and phosphorus originate from buffers.

The coating by the TiO_2_ composites is established by the two characteristic peaks at 4.5 and 5 keV corresponding to the TiKa and TiKb transmissions. There is also a profound increase in the heights of the silicon Ka peak at 1.73 keV thus confirming the presence of the silica xerogels. Traces of silver are only present in one case which is Black Finished Sheep leather treated with Ti-Si-PEI 25,000-Ag (Figure 4 orange). In contrast in the spectra of the leathers that were covered by simple silica xerogels (red spectra), incorporation of Ag Nps becomes more evident by the AgLI (2.8 keV,) AgLa (3 keV), AgLb (3.1 keV), and AgLg (3.5 keV) peaks. In this case, a considerable strengthening of the Si Ka is also observed. Finally, it is interesting to note and comment on the presence of titanium peaks in two of these samples given the fact that they are not treated by the titania powders. A possible explanation is the diffusion of the electrons due to the humidity present in the lower vacuum conditions that excites the neighboring samples. The same reason is probably responsible for the small iron peaks.

### 2.3. IR Spectroscopy

**Figure 5:**
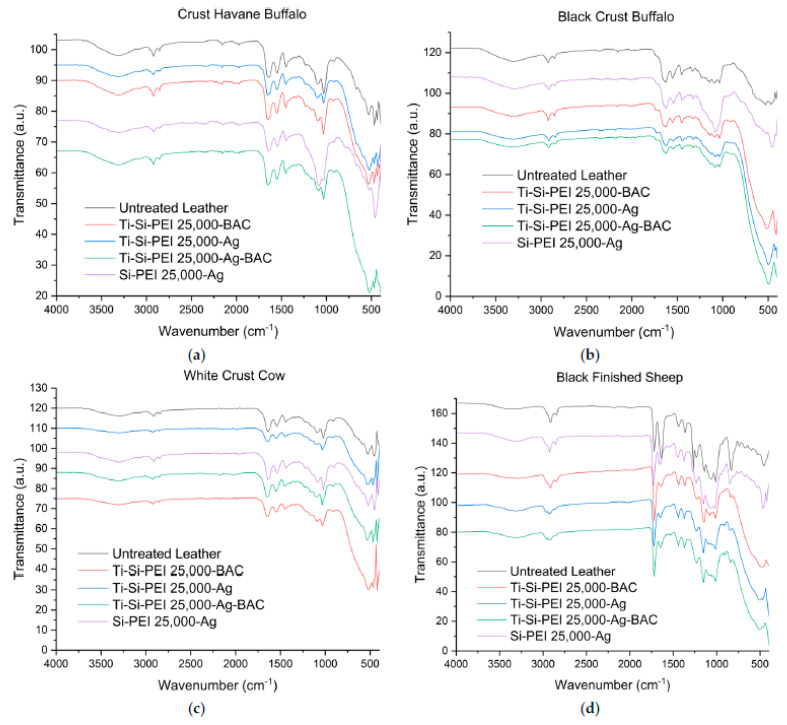
IR spectra of the four different leather types, (**a**) Crust Havane Buffalo, (**b**) Black Crust Buffalo, (**c**) White Crust Cow and (**d**) Black Finished Sheep before and after their treatment with each dispersion.

The FTIR spectra have contributed additional information on the organic composition of the leathers and the different dispersions sprayed on them (black spectra Figure 5). Buffalo and cow samples exhibit similar transmittance patterns. Collagen elastin and the other natural skin proteins can be identified by the C=O stretching band (Amide I) at 1649 cm^−1^, the N-H bending/C-N stretching (Amide II) band at 1548 cm^−1^, and the smaller protein β sheet and α helix peak at 1240 (Amide III) [56,57,58]. The triad’s third γ (CH2) bending band at 1450 cm^−1^ corresponds to the CH_2_ deformation of the proteins and lipids. The C-N antisymmetric stretch of the amino acids along with the bending band of secondary OH groups form the broad peak at 1100–1090 cm^−1^, while the neighbouring band at 1030 cm^−1^ corresponds to the symmetric C-N that overlaps the C-C stretching band. The hydrogen bonding network between amines hydroxyls and peptide groups is apparent from the broad band at 3300 cm^−1^, while the small band at 3080 cm^−1^ is due to the harmonic N-H stretching vibration (amide II). In the sheep counterpart, the major difference is the presence of a strong peak at 1720 cm^−1^ that corresponds to the C=O stretching vibration of non-ionized carboxylic acids and esters [59]. The amide I band is very strong while the amide 2 band is barely visible. The C-C band at 1000 cm^−1^ overlaps the symmetric C-N stretch and the C-O band at 1070 cm^−1^ overlaps the antisymmetric C-N analogue.

The presence of the silica xerogel on the leather’s surface is established by the characteristic pattern of strong bands: Si-O-Si stretch (1054 cm^−1^), Si-O-Si bend (793 cm^−1^), and rock (443 cm^−1^) [60]. Titanium dioxide powder inclusion in the dispersion composition is evident from the presence of the broad Ti-O stretching band with a maximum of about 475 cm^−1^ [61]. At this point, it is interesting to note that neither the BAC nor the silica bands are visible in the samples coated by the titania dispersions. This is an indication that a great percentage of the silica xerogel is indeed incorporated in the pores of the TiO_2_ ceramic.

### 2.4. Surface Hydrophilicity/Hydrophobicity—Water Wettability

**Figure 6:**
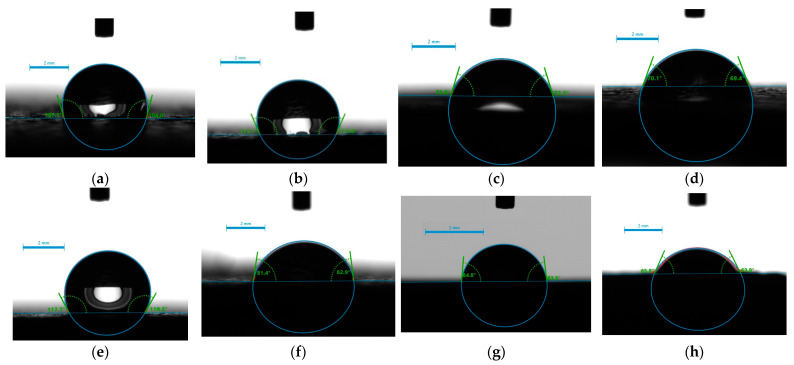
Water contact angles of (**a**) Crust Havane Buffalo, (**b**) Black Crust Buffalo, (**c**) White Crust Cow, (**d**) Black Finished Sheep, (**e**) Crust Havane Buffalo with Si-PEI 25,000-Ag, (**f**) Black Crust Buffalo with Ti-Si-PEI 25,000-Ag, (**g**) White Crust Cow with Ti-Si-PEI 25,000-Ag-BAC, and (**h**) Black Finished Sheep with Si-PEI 25,000-Ag (at the time, *t* = 0 s).

The affinity of the leather samples to water was assessed by the equilibrium contact angle of all studied samples (four untreated and twelve treated, as listed in Table 2) through the water contact angle (WCA) measurements. The two leathers 1 and 2 that came from buffalo are hydrophobic and have non-wetting surfaces. Water droplets stay stable, forming a contact angle between 105° and 113° (Figure 6a,b). Cow and sheep counterparts 3 and 4, respectively, exhibit substantial hydrophilicity (Figure 6c,d). The former is a material with a super-hydrophilic surface since WCA changes from 73° to 27.5°, and finally, the water drop is fully adsorbed after 0, 15, and 30 sec, respectively (see Table 2).

**Figure 7:**
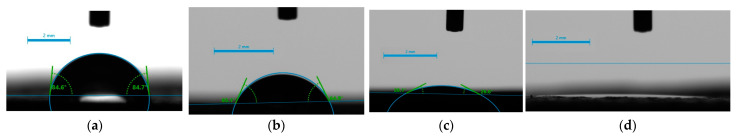
Water contact angles of White Crust Cow with Si-PEI 25,000-Ag at different time intervals. (**a**) *t* = 0 s; (**b**) *t* = 2 s; (**c**) *t* = 4 s; (**d**) *t* = 8 s.

From the data in Table 2 and Figure 6 and Figure 7, it is evident that in contrast to the silica xerogels formed into the pores [54], the application of silica dispersion does not enhance the hydrophilicity of the Crust Havane Buffalo sample (Figure 6e), whereas it has a significant effect on the Black Crust Buffalo counterpart. It rather delays water adsorption into the hydrophilic surfaces (Figure 7). The application of TiO_2_ dispersions enhances the hydrophilicity of the buffalo samples (Figure 6f). Impressively, there is a large synergistic effect between BAC and TiO_2_ that dramatically enhances the wettability of Black Crust Buffalo and renders its surface completely permeable (from 113° to 42° and 68° for Ti-Si-PEI 25,000-BAC and Ti-Si-PEI 25,000-Ag-BAC, respectively. Generally, this type benefited from all the additives. The wettability of the sample coming from cows (Figure 6g) is more or less retained through all processes, whereas the opposite applies to the sheep leathers except for the Si-PEI 25,000-Ag dispersion.

### 2.5. Antimicrobial Activity

#### 2.5.1. Disk-Diffusion Method

**Figure 8:**
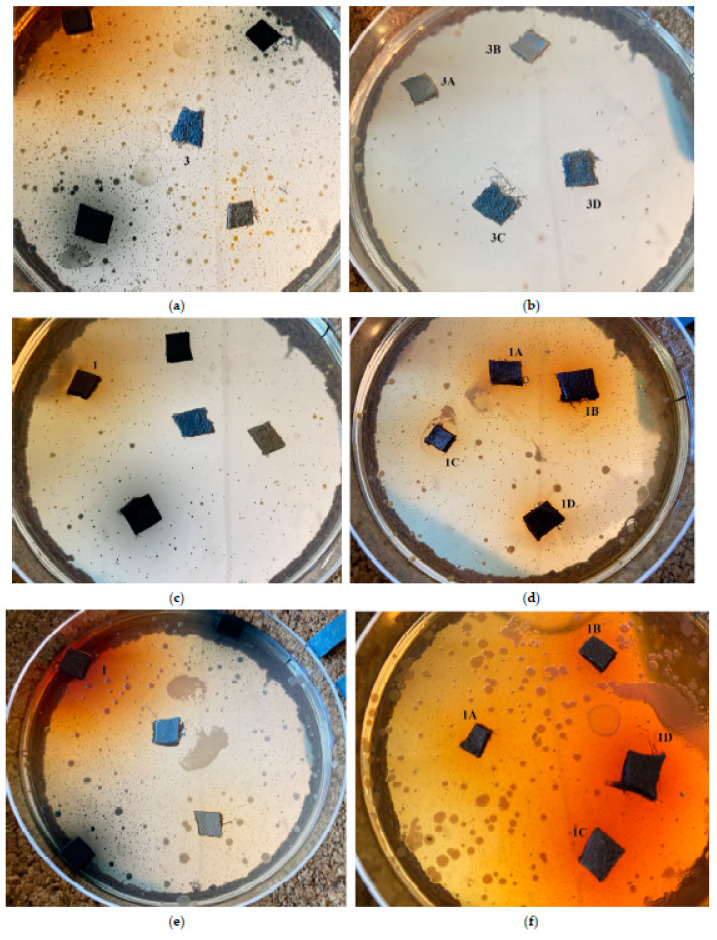

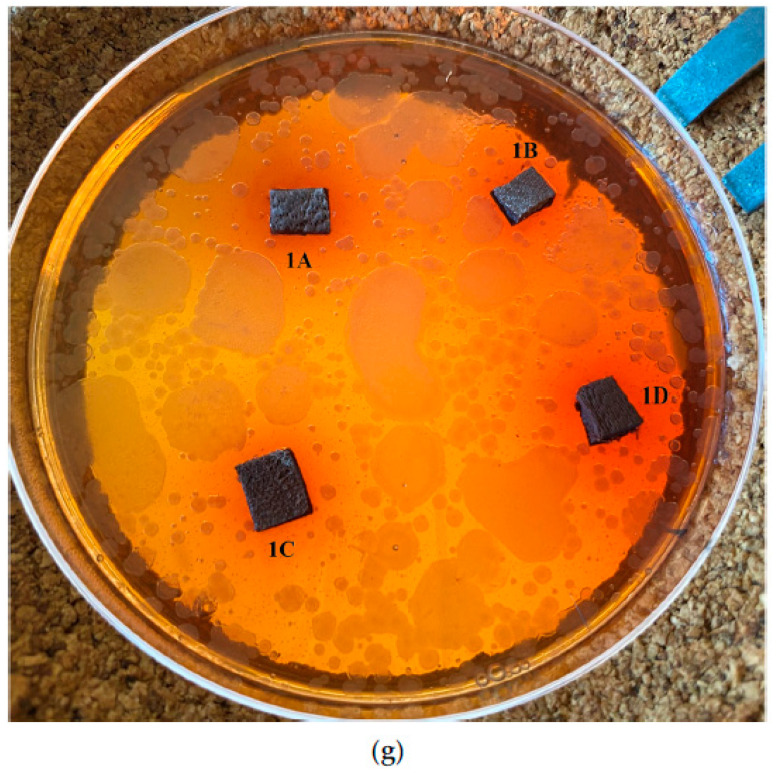
Representative Luria–Bertani (LB)-medium agar plates, indicating the propagation of *Escherichia coli* near White Crust Cow samples when untreated (**3**) (**a**) and when Ti-Si-PEI 25,000-BAC **3A**, Ti-Si-PEI 25,000-Ag **3B**, Ti-Si-PEI 25,000-Ag-BAC **3C**, Si-PEI 25,000-Ag **3D** dispersions are applied (**b**); *Staphylococcus aureus* near Crust Havane Buffalo samples when untreated (**1**) (**c**) and when Ti-Si-PEI 25,000-BAC **1A**, Ti-Si-PEI 25,000-Ag **1B**, Ti-Si-PEI 25,000-Ag-BAC **1C**, Si-PEI 25,000-Ag **1D** dispersions are applied (**d**); *Pseudomonas aeruginosa* near White Crust Cow samples when untreated (**3**) (**e**) and when Ti-Si-PEI 25,000-BAC **3A**, Ti-Si-PEI 25,000-Ag **3B**, Ti-Si-PEI 25,000-Ag-BAC **3C**, Si-PEI 25,000-Ag **3D** dispersions are applied (**f**) and after additional incubation (**g**).

To get a first assessment of the antibacterial potential of the dispersions and the diffusion of the active ingredients, a first testing was performed for three common bacteria: *Escherichia coli* (Figure 8a,b), *Staphylococcus aureus* (Figure 8c,d), and *Pseudomonas aeruginosa* (Figure 8e–g). As depicted in the indicative photos, leather samples covered by the various additives which displayed larger halos when bacteria proliferation was limited or non-existent (Figure 8b,d,f) than the untreated counterparts (Figure 8a,c,e). Interestingly, even after prolonged incubation, the coatings in some cases protected the substrates from contamination (Figure 8g).

**Figure 9:**
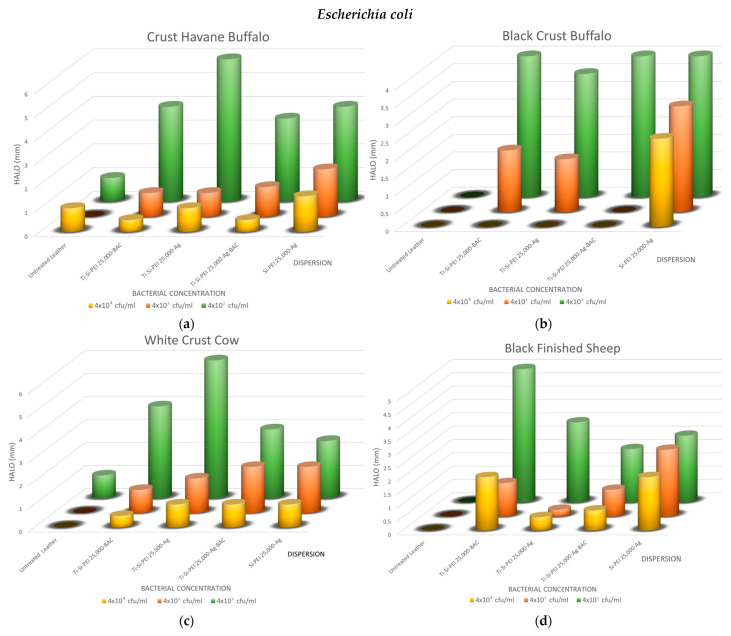
Propagation inhibition radii as a function of *Escherichia coli* concentration for all leathers and coatings of (**a**) Crust Havane Buffalo, (**b**) Black Crust Buffalo, (**c**) White Crust Cow, and (**d**) Black Finished Sheep.

**Figure 10:**
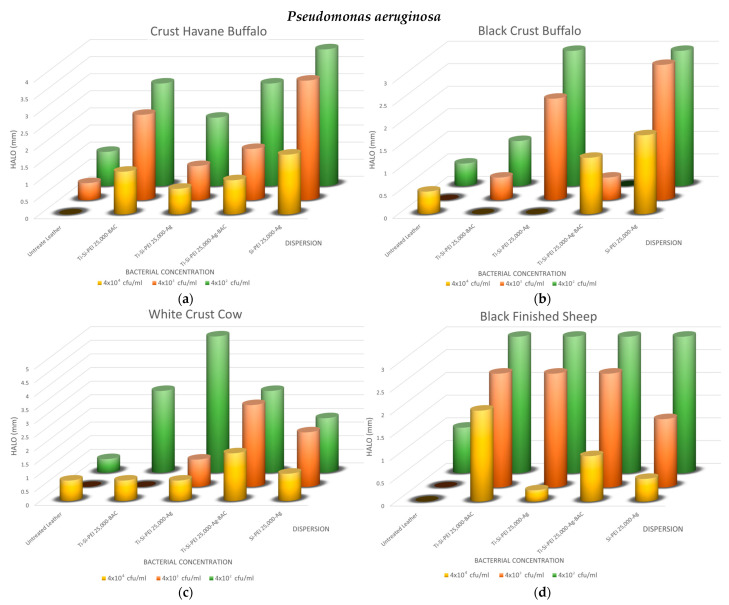
Propagation inhibition radii as a function of *Pseudomonas aeruginosa* concentration for all leathers and coatings of (**a**) Crust Havane Buffalo, (**b**) Black Crust Buffalo, (**c**) White Crust Cow, and (**d**) Black Finished Sheep.

**Figure 11:**
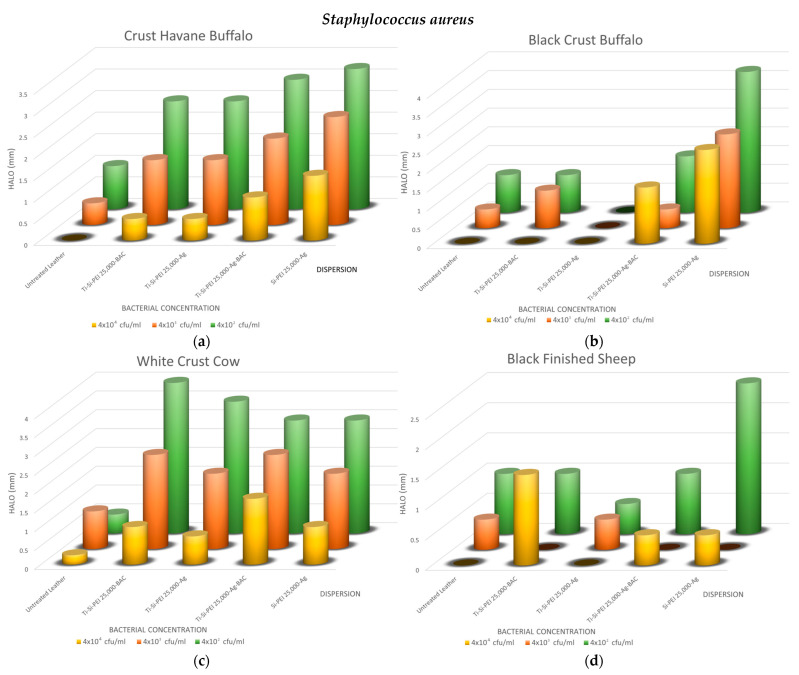
Propagation inhibition radii as a function of *Staphylococcus aureus* concentration for all leathers and coatings of (**a**) Crust Havane Buffalo, (**b**) Black Crust Buffalo, (**c**) White Crust Cow, and (**d**) Black Finished Sheep.

The overall results of the method are summarized in Figure 9, Figure 10 and Figure 11. As expected, the biggest halos are observed in the plates with the smaller concentrations. In all cases, the uncoated samples have no uncontaminated areas or very small ones, i.e., up to 1 mm. For the buffalo leather samples, the process that results in better antimicrobial action is the Si-PEI 25,000-Ag dispersion for all three microorganisms tested. This is the best-treated leather combination for the Gram-negative bacteria, *Escherichia coli* and *Pseudomonas aeruginosa*. There is an obvious explanation since it is the only treatment that does not contain the titania powder carrier. For this reason, the antibacterial silver nanoparticles are in this case in a higher ratio. The titania-covered leathers do display adequate protection against all bacteria with no dramatic hysteresis. Generally, silver nanoparticles may replace the controversial types due to its toxicity BAC. Notably, Black Finished Sheep leather when treated with Ti-Si-PEI-Bac nanoparticles also exhibits very good antibacterial activities for all tested concentrations for *Pseudomonas aeruginosa*. *Staphylococcus aureus*, the Gram-positive bacterium tested, also exhibits better growth inhibition when coming in contact with Ti-Si-PEI 25,000-Ag-BAC on White Crust Cow leather. The halos though in all cases were considerably smaller than those produced by the leather counterparts that were impregnated by the precursor silica gel formation solution at 1 × 10^5^ cfu/mL [52] or those generated by ampicillin that were in the area of 0.5 to 2 cm.

#### 2.5.2. Anti-Adherence Activity

**Figure 12:**
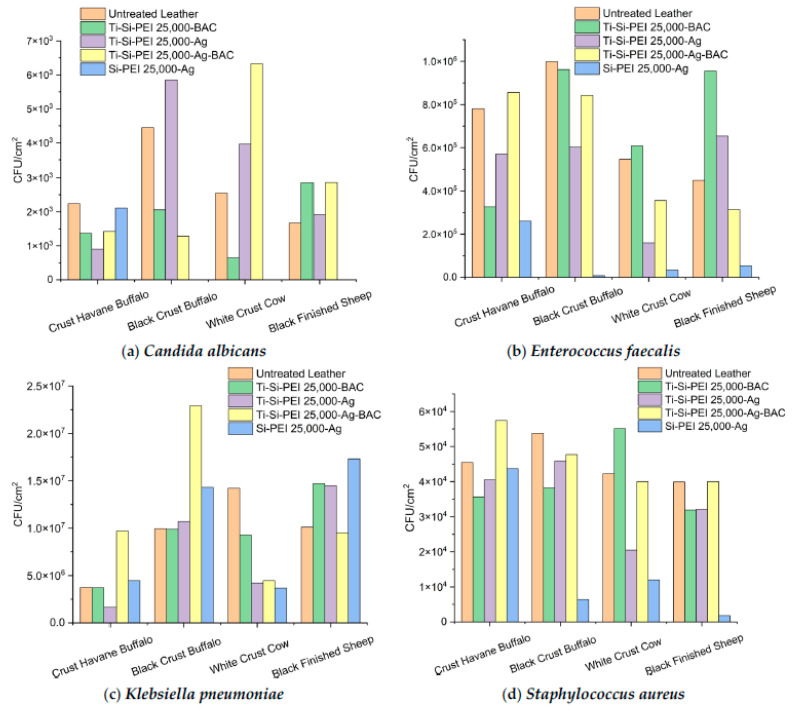
(**a**) *Candida albicans*, (**b**) *Enterococcus faecalis*, (**c**) *Klebsiella pneumoniae*, and (**d**) *Staphylococcus aureus* colonies adhered on the surfaces of the leather samples after two hours of incubation.

In a second series of experiments, we tested the capability of the different leathers to resist the attachment of a variety of bacteria (Figure 12). Complete protection was archived by Si-PEI 25,000-Ag against *Candida albicans* on the Black Crust Buffalo, White Crust Cow, and Black Finished sheep samples. Generally, the silica xerogels demonstrated good results in all samples for all microorganisms except Crust Havane Buffalo in combination with *Candida albicans*, *Staphylococcus aureus*, and *Klebsiella pneumoniae*. The latter gram-negative bacterium was insufficiently shielded on Black Crust Buffalo and Black Finished Sheep leathers. In contrast, Ti-Si-PEI 25,000-BAC presented good compatibility with Crust Havane Buffalo for all microorganisms, while Ti-Si-PEI 25,000-Ag inhibited the accumulation of most bacteria species onto the white crust surfaces. At this point, it is interesting to note that the combination of two different microbicide agents did not help prevent the adhesion of microorganisms onto the leather substrates.

#### 2.5.3. Antibiofilm Activity of Leather Samples

**Figure 13:**
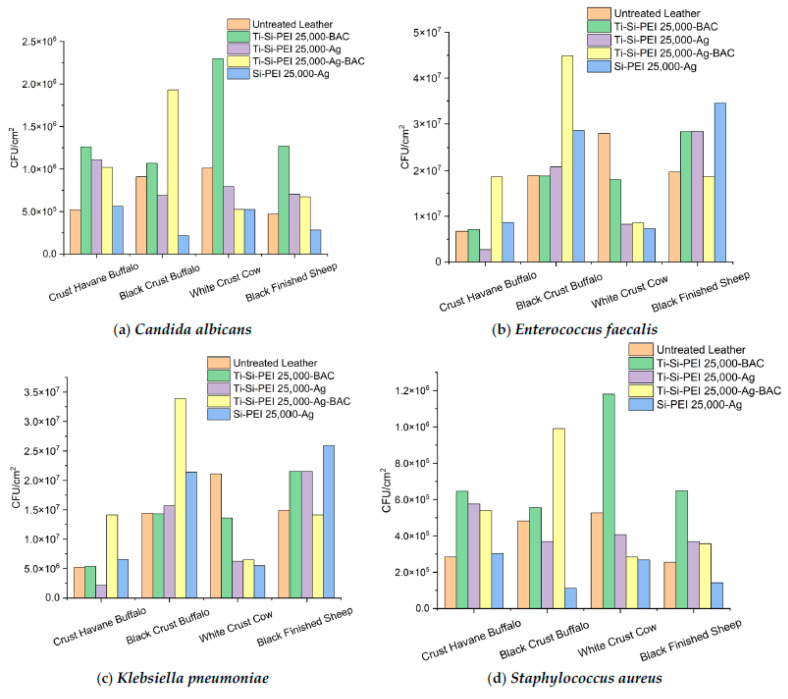
(**a**) *Candida albicans*, (**b**) *Enterococcus faecalis*, (**c**) *Klebsiella pneumoniae*, and (**d**) *Staphylococcus aureus* colonies adhered on the surfaces of the leather samples after 48 h of incubation.

In a final attempt to assess the antibiofilm potential of all tested dispersions, the incubation period of the microorganisms was prolonged to 48 h (Figure 13). In most cases, Si-PEI 25,000-Ag performed equally or better in comparison to the untreated counterparts. There was not a dramatic decrease in the observed colonies nonetheless. Dispersions based on titanium oxide powders did not produce statistically significant differentiations.

### 2.6. Difusion of Leather Components in Water, Stability of the Coatings

**Figure 14:**
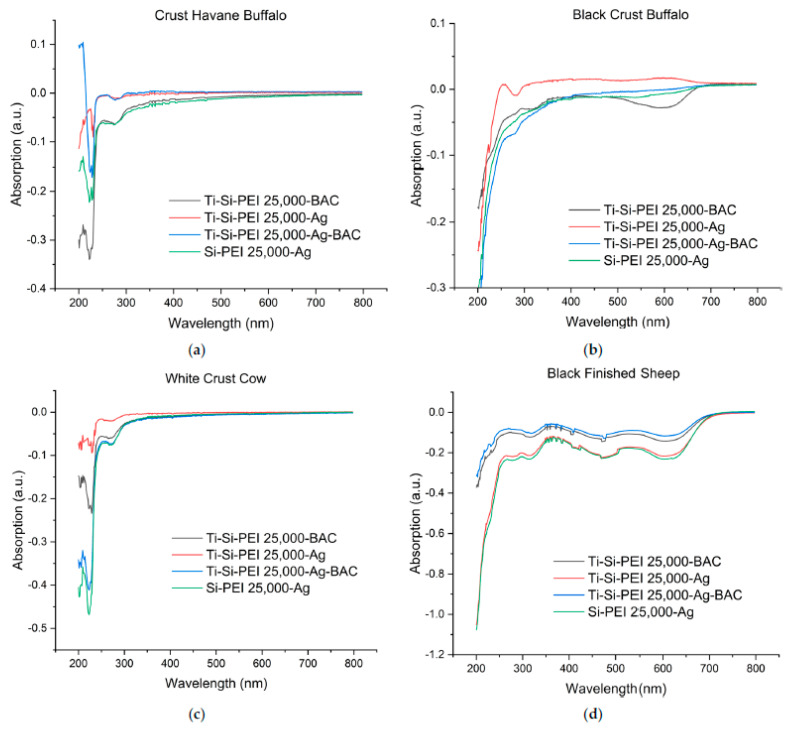
UV-Vis spectra of the supernatant solutions of (**a**) Crust Havane Buffalo, (**b**) Black Crust Buffalo, (**c**) White Crust Cow, (**d**) Black Finished Sheep coated with the different silica xerogel and titania/silica xerogel compositions after immersion in deionized water for a week. The respective supernatants of untreated leathers are used as controls.

For an estimation of the stability of the dispersion powders as well as all the other leather components, all samples (0.25 cm^2^) were immersed in deionized water and left under shaking for one week. UV-Vis absorption of the supernatants of each coated sample was then measured by using the supernatant of the respective untreated leather as a control (Figure 14). This direct comparison was performed due to the extensive diffusion of the components of the starting leathers. This in turn results in spectra having strong and wide peaks, particularly in the area of 300 to 200 nm that overlaps the smaller differences. In all the obtained spectra, the peaks shift downwards indicating that the concentration of absorbing components in the supernatants of the coated samples is smaller than that in the supernatants of the respective untreated leathers. Thus, the application of the xerogel powders decreases significantly the diffusion of the organic components and dyes of the tested leathers as observed in the case of the direct xerogel formation into leather pores [54]. Additionally, there is no peak at 420 nm, i.e., the characteristic absorption area of silver nanoparticles indicating that the latter are retained in the xerogels. Moreover, all the baselines were very close to zero suggesting the absence of scattering. This means that the silica xerogel and silica/titania xerogel powders applied remain adsorbed on the surface of the substrate and do not redisperse to water. As is additionally evidenced by the spectra, Si-PEI 25,000-Ag exercised the optimal effect, protecting more efficiently than the titania-based powders. This beneficial protection extends to the preservation of black color in the Black Crust Buffalo (Figure 14b) and Black Finished Leather (Figure 14d) in the area of 500–700 nm and can be a very useful property to limit the decolorization of all sorts of fabrics, leathers, and textiles observed after multiple washing times.

## 3. Conclusions

Two concepts have been proved from the experiments performed in the framework of this work. The spraying of silica xerogels containing active ingredients such as silver nanoparticles and/or BAC may adequately replace the formation of xerogels with similar compositions in leather pores. Furthermore, the antibacterial and antifungal activity is retained even if the silica xerogels are formed in the pores of a ceramic additive such as titania powder (TiO_2_). In both cases, however, there is a considerable decrease in the biocide and antibiofilm properties of the coating. In contrast, spraying silica xerogels does not always enhance the hydrophilicity of the substrates as did their incorporation in the leathers and not to the same extent. This disadvantage though can be negated by implementing the titania formulations. Moreover, all coatings were stable, did not redisperse in water, and protected the composition of the leathers, especially the black dyes from diffusion in the water. There is much additional research to be completed to obtain a commercially viable product. The optimum dispersion medium and the optimum quantity of silica xerogel into the titania particles need to be defined. The performance of the coatings may also be improved by increasing the quantity of the sprayed dispersion or by multiple spraying-drying cycles. The investigation of the modification of the porosity of the TiO_2_ carrier and the SiO_2_ filler by the gradual incorporation of the latter and various drying schemes presents substantial scientific interest. All this fine tuning combined with the employment of biomimetically formed TiO_2_ nanoparticles will provide environmentally safe composite materials for medical leathers and potentially for many more related applications.

## 4. Materials and Methods

### 4.1. Materials

Tetraethyl orthosilicate, (Si(OC_2_H_5_)_4_), ampicillin (C_16_H_19_N_3_O_4_S), and silver nitrate (AgNO_3_) were purchased from Sigma-Aldrich (Steinheim, Germany). PEI-25,000 (Mw = 25,000) (trade name: Lupasol WF) was obtained from BASF (Ludwigshafen, Germany), Benzalkonium chloride 50% solution ACTICIDE^®^ BAC 50 from Thor Company (Wincham Northwich, UK), trizma base (NH_2_C(CH_2_OH)_3_) from Research Organics (Cleveland, OH, USA), titanium dioxide (TiO_2_ RC 823) from CINKARNA CELJE, and 1-methoxy-2-propanol (CH_3_CH(OH)CH_2_OCH_3_) (PM) from SHELL Chemicals. Ultrapure water (18.2 M cm, Millipore Milli-Q system Millipore, Bedford, MA, USA) was used for the preparation of all aqueous solutions. All reagents were used without further purification.

### 4.2. Instrumentation

Leather samples before and after the treatment with the hybrid dispersions were examined by low-vacuum scanning electron microscopy (SEM) (FEI Quanta Inspect (FEI Hillsboro, OR, USA) microscope with a W (tungsten) filament) and energy-dispersive X-ray spectroscopy (EDS). They were also characterized by Fourier transform infrared spectroscopy (FTIR) (Nicolet Magna-IR ((Thermo Fisher Scientific, Madison, WI, USA) Spectrometer 550). Measurements of the dynamic contact angle (CA) between water droplets and the various leather surfaces were conducted on a Kruss DSA30S (Hamburg, Germany) possessing a range of 180° for surface tension, ranging from 0.01 to 2000 mN/m. The recording of the droplets’ digital images and the calculation of their contact angles were performed with the aid of the Advance-Kruss 1.5.1.0 software (Krüss Hamburg, Germany). The diffusion of both untreated and treated leather sample components was monitored by a Cary 100 UV–visible spectrophotometer I (Varian Inc., Palo Alto, CA, USA).

### 4.3. Pretreatment of Raw Bovine Buffalo and Sheep Hides

The bovine and buffalo hides used in the present work originated from the Greek region of Macedonia. Their surface area averaged 3.7 m^2^. Raw hides preserved with common salt were subjected to the standard treatment procedure for the production of wet-blue leather (soaking, liming, deliming, bating, pickling, and tanning by chromium (III) sulfate ([Cr(H_2_O)_6_]_2_(SO_4_)_3_). To then obtain crust, leather re-tanning using MgO and/or sodium aluminosilicate AlNa_12_SiO_5_., fat-liquoring, and dyeing followed. The sheep hides were obtained from the Peloponnese region in southern Greece. The average surface area was about 1 m^2^. A similar modification path was pursued. Small alterations of the method are limited to the utilization of slightly different reagents. In both cases, all the stages were accomplished in small laboratory drums. The final dry crust leather samples were used as substrates for the hybrid titania-silica xerogel dispersions.

### 4.4. Synthesis of Xerogels and Coating of Leathers

#### 4.4.1. Preparation of the Silica Xerogel-Silver Nanoparticle Dispersion (Si-PEI 25,000-Ag)

**Figure 15:**
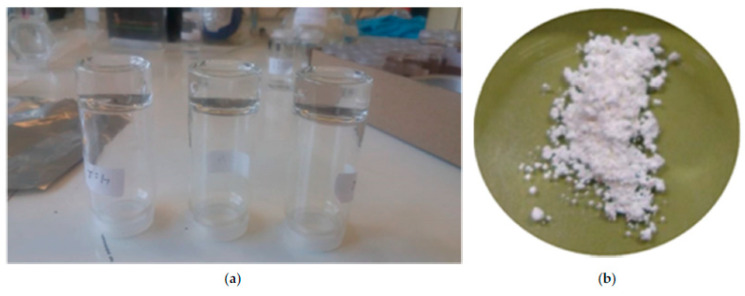
(**a**) Gels and (**b**) Xerogel powder prepared by hyperbranched PEI 750,000 and orthosilicic acid.

An amount of 12.5 mL of a 0.1 M AgNO_3_ solution was added to a 50 mL solution of hyperbranched PEI (Mw 25,000, 40 mM, in primary and secondary amino groups). After about an hour, the color of the mixture turned to light yellow indicating the beginning of the Ag Nps formation. The conclusion of silver nucleation occurs after 8 days when the solution becomes reddish brown [45]. For the preparation of hydrogels, 2.08 gr of tetraethoxysilane were hydrolyzed by a 5 mM HNO_3_ aqueous solution. The resulting 10 mL of 1 M orthosilicic acid solution was mixed with an equal quantity of the silver-hyperbranched PEI solution, and the pH was adjusted to 7.5 with a trizma base. Gelation was observed after two hours as indicatively shown for samples of orthosilicic acid and PEI (Figure 15a), and drying to xerogel powders (Figure 15b) was carried out by gentle heating at 60 °C for 5 days followed by a final drying stage under vacuum and over P_2_O_5_.

#### 4.4.2. Preparation of the Titania-Silica Xerogel-Silver Nanoparticle Dispersion (Ti-Si-PEI 25,000-Ag)

The method is the same as before. The gel precursor solution (20 mL) is added to 30 g of TiO_2_ to be adsorbed by the ceramic powder and form a solid paste. In this way, the gelation occurs in the pores and the xerogel is incorporated into the inorganic substrate. The gelation-drying procedure is the same as described before.

#### 4.4.3. Preparation of the Titania-Silica Xerogel-BAC Dispersion (Ti-Si-PEI 25,000-BAC)

An amount of 10 mL of the 1 M orthosilicic acid solution prepared as described before was mixed with an equal quantity of a PEI 25,000 solution (40 mM, in primary and secondary amino groups). After the pH adjustment, as described before, they were added to 30 g of TiO_2_. After the already described gelation and drying processes, 20 mL of the BAC 50 solution was added to the resulting powder and the drying procedure was repeated once more.

#### 4.4.4. Preparation of the Titania-Silica Xerogel- Silver Nanoparticle-BAC Dispersion (Ti-Si-PEI 25,000-Ag-BAC)

The method is similar to that previously described in Section 4.4.3 with only a difference. Instead of the simple PEI 25,000 solution, a silver-hyperbranched PEI 25,000 solution was used. The latter was prepared as described in Section 4.4.1. In a standard industrial process, all powders were dispersed to 45 g PM and then sprayed with a spray gun (Springfield Leather Company, Springfield, MO, USA) attached to an air compressor onto the leather coupons (20 mL/cm^2^ of leather). The PM molecules of the solvent play a multifunctional role as they act as carriers as well as lubricants that help the dispersion particles to penetrate leather pores and binders. They form hydrogen bonds with the silanol and amino groups of the xerogel and enhance in this way the density of the hydrogen bond network that forms between the coating and the molecules of collagen, elastin, and the other leather proteins thus stabilizing the powders onto the surface.

### 4.5. Disk-Diffusion Method

The four different kinds of leather that were submitted to the treatment with each dispersion (Ti-Si-PEI 25000-BAC, Ti-Si-PEI 25000-Ag, Ti-Si-PEI 25,000-Ag-BAC, and Si-PEI 25000-Ag) were evaluated along with uncoated counterparts. All samples were cut into squares (0.5 cm × 0.5 cm) and sterilized for 30 min by UV light (λ = 254 nm) (15 min on each side). Gram-negative as well as the Gram-positive bacteria were chosen as model microorganisms for this study. *Escherichia coli* ATCC 25922, *Staphylococcus aureus* ATCC 29213, and *Pseudomonas aeruginosa* ATCC 27853 were cultivated overnight at 37 °C with shaking at 200 rpm in Luria–Bertani (LB) medium. Dilution in LB agar (0.8% *w*/*v*) then followed up to final densities between 4 × 10^2^ and 4 × 10^4^ CFUs/mL and spreading in LB agar. The leather specimens were brought into contact with the bacteria. The antibiotic ampicillin was used as the positive type and deionized water as the negative control for water. After a second overnight incubation period at 37 °C, the diameters of the halos around the samples with minimal or no bacterial growth were counted.

### 4.6. Anti-Adherence Properties of Leather Samples

Triplicate samples of leathers treated by hybrid silica and titania xerogel dispersions were introduced in 6-well cell culture plates with 4 mL of bacterial inoculum (5 × 10^5^ CFU/mL). After 2 h incubation, the supernatants were isolated, the leather coupons were submitted to washing with 1× PBS solution and sonication for the detachment of the bacteria that were adhered onto their surfaces, and colony counting followed. The antibiofilm testing was conducted by the same process as described in the previous chapter for the anti-adherence experiments with an incubation time of 48 h.

## Figures and Tables

**Table 1:** Classification of the leather types and treatment methods of this study.

Dispersion				
Leather Type	Crust Havane Buffalo	Black Crust Buffalo	White Crust Cow	Black Finished Sheep
Blind (untreated)	1	2	3	4
Ti-Si-PEI 25,000-BAC	1A	2A	3A	4A
Ti-Si-PEI 25,000-Ag	1B	2B	3B	4B
Ti-Si-PEI 25,000-Ag-BAC	1C	2C	3C	4C
Si-PEI 25,000-Ag	1D	2D	3D	D4

**Table 2:** Water contact angle properties of all studied leather samples.

Sample Name	*t*_(*s*)_ = 0	*t*_(*s*)_ = 15	*t*_(*s*)_ = 30	*t*_(*s*)_ = 60	Comment
1	104.5	105.6	104.8		stable drop
2	113.4	104.9	109.7		stable drop
3	73.05	27.5	disappeared		fully adsorbed
4	69.75	64.35	62.85		unstable drop
1A	99.05	88.0	83.5		unstable drop
2A	41.85	19.3	disappeared		fully adsorbed
3A	65.0	37.3	27.5	disappeared	fully adsorbed
4A	102.35	88.4	83.35	84.65	unstable drop
1B	96.75	86.75	81.2	78.65	unstable drop
2B	82.15	10.8	disappeared		fully adsorbed
3B	58.85	37.7	disappeared		fully adsorbed
4B	95.4	87.5	85.65	85.45	unstable drop
1C	110.8	104.5	99.1		unstable drop
2C	67.9	52.5	49.1	25.8	adsorbed
3C	84.15	67.0	43.35	27.35	adsorbed
4C	84.75	75.5	74.6	73.5	unstable drop
1D	118.6	119.2	119.2		stable drop
2D	78.45	67.6	60.5		unstable drop
3D	84.65	65.0	27.35	disappeared	fully adsorbed
4D	64.85	50.55	54.0	44.3	adsorbed

## Data Availability

Data is contained within the article.
